# What’s love got to do with jealousy?

**DOI:** 10.3389/fpsyg.2023.1249556

**Published:** 2023-09-28

**Authors:** Ana Maria Fernandez, Maria Teresa Barbato, Belen Cordero, Yvone Acevedo

**Affiliations:** Laboratorio de Evolución y Relaciones Interpersonales, Universidad de Santiago de Chile, Santiago, Chile

**Keywords:** affect, pair bonds, evolution, attachment, mating

## Abstract

Romantic love and jealousy seem antagonistic, but the expression of both emotions have evolutionary functions that can go in the same direction of maintaining a relationship. Considering natural selection designed adaptations to solve the problems surrounding reproduction, then love and romantic jealousy are emotions aimed at staying cooperative for a period of time, where love solves the adaptive challenges of promoting pair bonding, cooperation, and protecting offspring; and jealousy is triggered by a threat or the loss of a valuable cooperative relationship, either on behalf of descendants in need of resources, or a close romantic bond. Consequently, understanding love and romantic jealousy points in the same adaptive functional domain of protecting a romantic pair bond. Specifically, love can be comprehended in two different ways and in regard to jealousy. First, conceiving love as the attachment to significant others one develops throughout lifetime, and secondly, it contemplates affective dependence. Results from a sample of single and committed individuals (*n* = 332) show the predicted positive correlation between attachment and jealousy as stable traits, consistent with previous literature. In addition, there is a non-significant and low correlation, respectively, between attachment and love as a measure of dependence. Furthermore, in the single participants group, jealousy was associated with love. The discussion emphasizes the need for expanding a functional account of love and jealousy as complementary emotions of our human affective endowment. Finally, it would be informative to study attachment as a relational trait and love as a specific affection for a romantic partner that could be manipulated to elucidate the functional design of jealousy.

## 1. Introduction

The study of love encompasses different perspectives from diverse disciplines, such as anthropology, genetics, biology, neurobiology, and psychology (De Boer et al., 2012; Carter and Porges, 2013; Cacioppo and Cacioppo, 2015; Tobore, 2020; Langeslag, 2022). There is general agreement in describing love as a complex emotion, having multiple expressions (Hatfield and Sprecher, 1986) and favoring long-term mating (Sorokowski et al., 2017). However, the experience of love is so broad that several lines of research are needed to understand its origin, function, and the mechanisms underlying this phenomenon. Love has numerous functions such as contributing to mate choice, courtship, sex and pair bonding (Bode and Kushnick, 2021), among others, and one of the most relevant is that it uniquely endows our species with evolutionary advantages (Frank, 1988; Gonzaga and Haselton, 2008; Durante et al., 2016). Indeed, love functions as a commitment mechanism that facilitates pair bonding (Miller and Todd, 1998; Fletcher et al., 2015; Ein-Dor and Hirschberger, 2016).

Pair bonding is a crucial process associated with love, which has been described as a functional feature present in most mammals, with specific neuroendocrine activation, along with the promotion of mother-infant attachment (Harlow, 1958; Bales et al., 2021). To better understand the engagement function of love and pair bonding from an evolutionary perspective, the neurophysiological maturation of the mammalian brain exhibits a phylogenetic link to social involvement and attachment behaviors (Porges 1998; Cacioppo et al., 2015). Additionally, there are biochemical mechanisms of social engagement regulation, where molecules such as oxytocin and/or vasopressin are directed to facilitate pair bonding (Porges 2011; Carter and Porges, 2013; Perry-Paldi et al., 2019), which has also been described as a mechanism underlying human attachment (Feldman, 2016).

Attachment theory (Bowlby, 1982; Fraley, 2019; Thompson et al., 2022) explains how the pair bond established with a primary caregiver early in life influences one’s future relationships with the world, including interactions with others and the quality of affective relationships, such as engagements in couples (Hazan and Shaver, 1987). In general, research on romantic love in adults highlights and captures most of the adaptive characteristics of mother-infant attachment when adults establish a romantic pair bond (Hazan and Shaver, 1987; Shaver and Hazan, 1988; Fisher, 1998). According to Fletcher et al. (2015), romantic love can be conceived as an “evolved commitment device” with the ultimate function of motivating the potential reproductive partner to maintain sexual exclusivity long enough to procreate and raise offspring (Hazan and Diamond, 2000). Thus, kindness, empathy, care and feelings of warmth, which are typical of early pair bonding, are also present in romantic attachment (Fraley, 2019); but romantic bonding also compromises the lust or sexual attachment system (Shaver and Hazan, 1988; Fisher, 1998). Therefore, the study of attachment has become very relevant to understanding the nature, building of, and maintenance of couple bonds (Mikulincer and Shaver, 2007). Consequently, the formation of a pair bond may be linked to the origin of love, as it serves as an ubiquitous commitment mechanism.

The empirical evidence suggests that romantic love conveys diverse proximate components and is influenced by individual factors (Perry-Paldi et al., 2019). Elements such as intimacy, passion and commitment are universal influences of the love experience (Finkel et al., 2017; Sorokowski et al., 2017; Neto, 2023). On the other hand, individual differences, such as gender, age, and cultural modernization, can impact the experience of romantic love (Feybesse and Hatfield, 2019; Sorokowski et al., 2023). The significance of relationship satisfaction, effective communication, and mutual support (Yoo and Joo, 2021), indicates that romantic love is a multifaceted phenomenon involving multiple constituents, social and individual factors (de Munck et al., 2016).

In the search for a better comprehension of love, it has been suggested that this emotion can be described as feelings of affection, dependence, liking and caring - “a state of intense longing for union with another” (Hatfield et al., 2012, p. 144), with several theories aiding to understand love, such as attachment theory (Bowlby, 1982), the triangular theory of love (Sternberg, 1986), and interdependence theory (Baumeister and Vohs, 2007), to name a few main ones. Consequently, commitment and dependence on one another is a factor that was first identified in the conception of investment and exchange of benefits as crucial components of love (Kelley and Thibaut, 1978; Joel et al., 2013), as well as the perception of the loved one as part of the self (Aron et al., 2022). In this context, measures of love have been based on observational strategies, implicit associations through correlations of scales, and self-reports of the different hypothesized components of love (Graham, 2011), for example. So far, the methods used to assess love have relied on self-reported measures encompassing various definitions, such as lifelong attachment, intimacy, compassion, and dependence (Fabella, 2023). Nonetheless, when defining love as attachment, Hudson and Fraley (2017) suggests diverse levels of perceived intimacy and dependence are associated with attachment styles, which may hinder functional hypotheses about this emotion. Overall, there is no consensus on the measures employed to evaluate love, and how we quantify this emotion relies on the theoretical framework employed (see Hatfield et al., 2012, for example).

Attachment has a direct influence on the cognitive control of dyads in love, enhancing their ability to regulate primary emotions and thoughts (Langeslag and van Steenbergen, 2019). Studies looking at individual differences in attachment styles when assessing characteristics like dependence or closeness, have found that individuals with anxious attachment tend to require more time and affection to perceive they are loved by a partner (Hudson and Fraley, 2017). Similarly, Barbaro et al. (2021) reported certain attachment orientations (for example, anxious or avoidant) are related to mate-retaining behaviors, like controlling the partner across time. Individuals who develop security in their attachment, tend to have more satisfying interpersonal relationships and romantic partners, while “the most emotionally powerful experiences that people have in their lives derive from the development, maintenance, and disruption of attachment relationships” (Fraley, 2019, p. 419).

Following this same line of assessing the function of love and attachment, jealousy has been studied as an emotion that motivates the protection of a valued relationship (Mathes, 1986; Buunk, 1997; Neal and Lemay, 2014). Romantic jealousy has been conceived as an affective reaction specifically designed for the protection of close attachment bonds (Fernández, 2017; Fernández et al., 2022), and as far as romantic relationships are concerned, it is an emotion aimed at the protection of pair bonds (Fletcher et al., 2015).

However, much of the research on romantic and sexual jealousy has mainly been based on the use of hypothetical scenarios (Buss, 2018) and retrospective accounts of infidelity (Schützwohl, 2008). For example, using scenarios present imaginary situations of romantic betrayal (Buss et al., 1999; Sagarin et al., 2012; Bendixen et al., 2015), and methods like movie watching (Fernández, 2012) and reading stories about infidelity (Sabini and Silver, 2005) have been employed. In general, fictional scenarios allow participants to mentally recreate extradyadic partner involvement, which are then linked to forced-choice questions. These accounts present two fictional cases, such as sexual or romantic infidelity, and the subjects are forced to choose which situation generates more jealousy (Harris, 2004). Thus, the ecological validity of these experiments depends on variables that may not be controlled for in the experimental designs. For instance, experiencing partner infidelity in real life can significantly influence the experience of jealousy (Buunk and Fernandez, 2020), and watching movies or reading stories may elicit a specific jealousy response when subjects do or do not engage with the situation (Strout et al., 2005).

In general, research on jealousy has focused on identifying sex differences between emotional and sexual types of infidelity, while contextual differences in terms of partner investment and sample type have been looked at more seldom (Scelza et al., 2019). Exploring cultural differences in jealousy helps understanding jealousy as an adaptive reaction to changes in resource diversions in a given environment. Therefore, current work supports the importance of considering other variables, such as parental investment and paternity uncertainty, which are associated with an enhanced jealousy response (Edlund et al., 2019).

Along these lines, the study of jealousy has been linked to improved measurement of sex differences between sexual and emotional infidelity, using methodological innovations. For example, using economic games to examine if the allocation/reception of resources from a rival evokes this emotion (Barbato et al., 2018), and the presentation of spatial arrangements between the subject, his or her partner and a potential rival to assess jealousy, through certain threats (Schützwohl et al., 2011). Therefore, a similar approach to study love may aid in the precision of its measurement and assessment.

From an evolutionary perspective, cognitive biases in the form of adaptive design were shaped by natural selection to solve reproductive problems (Cosmides and Tooby, 2013), as there are biological and reproductive costs associated with exclusive resource allocation for offspring rearing (Buss, 2013; Fernández, 2017). In this regard, the design of love and jealousy may be linked to the creation of a mechanism for encouraging dependence and protection of the benefits that commitment and romantic engagements bring about (Conroy-Beam et al., 2015; Fletcher, 2015). In other words, love could serve as a promoter of altruistic prosocial behaviors associated with the bonding partner, resulting in high benefits to a dyad (Buss, 2007; Fletcher et al., 2015), while jealousy enables the retention and monopolization of the bond in potential infidelity scenarios (Buss et al., 1992; Harris, 2003).

Consequently, the pair bonding present in romantic love aids in the provision of psychological resources advocating care and reproductive success (Buss, 2019). Indeed, romantic love is a bond conveying the provisioning of resources which brings about an implicit assumption of exclusivity, through sexual and emotional fidelity toward the partner. In this sense, it is posited that for there to be a commitment triggered by romantic love the reproductive success of the individual in a potentially procreative bond requires an interdependence of fitness; where the ability to promote the genes of one person depends on the other one (Aktipis et al., 2018). The maintenance of long-term benefits through the commitment promoted by romantic love, implies that each partner must push the other to obtain benefits from acts of reciprocity for their common reproductive goals, and achieving a reciprocal balance (Cosmides and Tooby, 2013; Conroy-Beam et al., 2015). Hence, there is not only the commitment triggered by the emotion of romantic love, but there may be other emotions such as jealousy, which ensure that the benefits achieved by the initial commitment, are maintained over the long term. For this reason, from an evolutionary point of view suspicion about the probability of losing benefits or commitment by the cooperating partner must be paramount for maintaining the valued bond (Buss and Haselton, 2005; Foster et al., 2014). So, understanding love and jealousy is in the same adaptive functional direction of protecting human pair bonding.

According to attachment theory, affectionate bonding and distinctive valuation of significant others emerges throughout the life cycle, expanding from the internalization of childhood experiences into friendships and romantic attachment (Bohn et al., 2023). Early infant bonding facilitates adaptive fitness by motivating caring and safety of infants, generating an affectionate engagement prompting the child to seek proximity, sensory contact, and comforting from the primary caregiver (Bowlby, 1982; Thompson et al., 2021). Indeed, “the attachment system evolved to protect infants from danger by keeping them close to the mother” (Hazan and Shaver, 1987, p. 512), particularly in the ancestral environment (Hrdy, 2009). Attachment brings enormous psychosocial advantages to humans (Harlow, 1958; Hazan and Diamond, 2000), generating an emotional base of felt security, love and dependence, and reducing anxiety in times of distress (Fraley, 2019).

Attachment also plays a crucial role in regulating stress and promoting emotional well-being among individuals. It facilitates co-regulation within dyads, fostering a sense of security, reducing separation distress, and fulfilling the need for affectionate physical contact (Zeifman, 2019), which has been recently evidenced cross-culturally (Sorokowski et al., 2023). This emotional system, connected to social defense theory, has biochemical characteristics that enable individuals to navigate complex social environments and enhance their survival (Ein-Dor and Hirschberger, 2016). Furthermore, romantic attachment brings about dyadic benefits by serving as a mechanism for mate choice and fostering courtship attraction. It is an integral part of the adult attachment system, ensuring that parents stay together to raise their offspring effectively (De Boer et al., 2012). In this way, attachment not only promotes individual well-being, but it also contributes to the stability of romantic relationships.

It is worth noting the connection between attachment and jealousy has been extensively documented, with attachment anxiety being a strong predictor of jealousy (Rodriguez et al., 2015; Barbaro et al., 2016; Güçlü et al., 2017). Specifically, individuals with an anxious attachment style are more prone to experiencing anxious jealousy, while those with an avoidant attachment style are more likely to experience reactive jealousy (Buunk and Fernandez, 2020).

Attachment in general, can be conceived as a promoter of commitment, providing emotional security and satisfying affective needs in romantic partners (Ein-Dor and Hirschberger, 2016; Feldman, 2016; Buss, 2017). Attachment styles contribute to individual differences in the formation of feelings of security, creating a bond of dependence and fear of loss (Attridge, 2013). Jealousy, in this sense, plays an important role in understanding the protection of this affective bond (Buss, 2018; Buunk and Fernandez, 2020). From an evolutionary perspective, jealousy arises in response to the suspicion of losing a partner to a rival, considering the important benefits of long-term attachment (Schmitt and Buss, 2001; Buss and Haselton, 2005; Foster et al., 2014). Furthermore, attachment theory provides valuable insights for recognizing jealousy, particularly in relation to individuals with anxious attachment who express higher levels of trait jealousy compared to those with secure attachment (Marshall et al., 2013; Richter et al., 2022). Hence, attachment and jealousy are directed at the same end of facilitating romantic engagement. But, as Fernández (2017) revised, jealousy is specifically aimed at avoiding the diversion of partner resources that are beneficial in terms of fitness for both members of a romantic dyad.

In the present study, attachment was assessed, as well as love with independent measures of affective dependence. These were then correlated with subjective indicators of jealousy. Considering the functions of love and jealousy described in the literature, which suggest a common evolutionary purpose of promoting commitment, it was predicted that the function of love and jealousy go in the same direction of maintaining the benefits of a romantic relationship.

Accordingly, it was specifically anticipated that:

- In general, behaviors that trigger higher levels of jealousy are typically associated with the perceived risk of losing the bond and potential resources. Therefore, anxious attachment would be positively correlated with jealousy, as individuals seek to protect their romantic bond. In consequence, levels of jealousy and attachment will exhibit a positive correlation, stemming from their shared adaptive function.
- On the other hand, measures of love related to dependence would reflect characteristics of this emotion that may be associated with functions other than being a key to resource commitment. As a result, jealousy should not correlate with these particular characteristics. So, it was anticipated that levels of jealousy and the measure of love would not correlate, given they represent different facets within the domain of love and interpersonal relationships. Hence, while both variables may share certain commonalities, they also represent distinct aspects of attachment and interpersonal relationships.

## 2. Materials and methods

### 2.1. Participants

The complete research involved 332 Chilean people, who were recruited through social networks and took part in two studies. The first sample included 123 individuals (M age = 27.9, SD = 9.92, 67% female), with 47.2% of them indicating that they were not involved romantically. The second sample comprised 209 committed individuals (M age = 25.9, SD = 5.87, 67% female).

### 2.2. Measures

*Jealousy* was assessed by a single self-report question asking “how jealous you are?” (not jealous at all) to 7 (morbidly jealous), which has been previously used by Massar and Buunk (2010) and our laboratory in Chile (see Fernández et al., 2022). In an experimental sample of 48 participants (see Barbato et al., 2018), this item had a partial correlation with Buunk’s (1997) 15-item jealousy scale, of *r* = 0.46, *p* < 0.001 for reactive, *r* = 0.41, for anxious, and *r* = 0.58 for preventive jealousy (*p*s < 0.001, large effect size).

*Brief Spanish version of the experiences in close relationships, ECR* (Guzmán et al., 2020), is a widely used measure of anxious and avoidant romantic attachment with an observed reliability of Mcdonald’s ω = 0.82 and ω = 0.84, respectively.

*Attachment anxiety* (Fernández and Dufey, 2015) was measured using only the dimension of anxiety of Collins’s (1996) adult attachment scale revised (Mcdonald’s ω = 0.89).

*Dependance* (Attridge et al., 1998), is the degree of psychological and emotional dependence expressed toward the current partner, which was conceived as a measure of “love,” reaching an observed reliability of Mcdonald’s ω = 0.90.

### 2.3. Procedure

Participants were recruited through social networks. The samples completed the measures online. All participants signed an informed consent according to the ethical principles of APA, and responded to a sociodemographic questionnaire, measures of jealousy, attachment anxiety (Collins’s and ECR, in the singles and committed sample, respectively), love (dependence). Each study was approved by the Institutional Ethics Committee of the author’s University.

### 2.4. Data analyses

Descriptive statistics, correlations and regression analyses between the variables were estimated using Jamovi (2021).

## 3. Results

Our first prediction was partially supported (see Table 1) with a significant positive association between attachment and jealousy in both samples, and a non- significant correlation of love and attachment found for the single sample. The correlation of love and anxious attachment was low but significant.

**Table 1:** Descriptive statistics and correlations by sample.

	Mean	SD	2	3
Sample 1 (*n* = 123)				
Jealousy	2.71	0.95	0.24**	0.22*
Attachment anxiety	3.01	1.11		0.14
Love	4.84	0.83		
Sample 2 (*n* = 209)				
Jealousy	3.33	1.53	0.57***	0.16*
Anxious attachment	3.56	1.53		0.22**
Love	4.39	0.51		

*p < 0.05, **p < 0.01, ***p < 0.001.

Secondly, multiple regression analysis yielded jealousy, as the only significant predictor of love (*t* = 2.13, *p* = 0.035) in the single sample (*F*_2,120_ = 3.54, *p* = 0.032, *r*^2^ = 0.048). While anxious attachment (*t* = 2.35, *p* = 0.020) uniquely predicted love (*r*^2^ = 0.050) in the committed sample (*F*_2, 206_ = 5.45, *p* = 0.005).

## 4. Discussion

The assessment of the adaptive function of love and jealousy was studied by examining if specific traits associated with love, such as attachment were correlated to jealousy. It was predicted that romantic love could underlie attachment and jealousy, having the evolved function of protecting attachment from situations or rivals that may pose a threat to a reproductive bond (Buunk, 1997; Buss, 2018).

The first prediction was confirmed as anxious attachment was associated with jealousy. More specifically, in the single sample, the dimension of anxiety was associated with jealousy, and in the committed individuals, attachment anxiety and jealousy were positively correlated. These findings support the idea that attachment and jealousy might operate in conjunction, sharing a similar adaptive function.

Furthermore, contrary to our second prediction, a positive association between jealousy and love emerged in the single participants’ sample. Despite this, no significant correlation was found between attachment and love, suggesting the existence of unequivocal elements within love, conceived as dependence, which may be immersed in the experience of love. In general, these outcomes reinforce the notion that love and jealousy operate in tandem, reflecting a shared functional logic centered around close relationship protection. Moreover, these results align with traditional research that links love to indicators of jealousy, alongside psychological factors such as insecurity and low self-esteem (Mathes and Severa, 1981; White, 1981; Richter et al., 2022).

However, when looking at the prediction of love from attachment anxiety and jealousy, we had different results for the single and committed samples. Jealousy was the only variable that predicted love in the first sample, and attachment was the only predictor of love for the second sample. This may be indicative, that when people imagine, but do not have an actual committed romantic bond, they may attribute more jealousy to feelings of love, independent of their anxious attachment. But when committed individuals report on their romantic bond to an actual partner, anxious attachment does explain love, above and beyond jealousy.

Furthermore, characteristics such as romantic dependence describe alternative ways of experiencing love which do not appear to involve jealousy, and may be idealized in people that are not actually in a committed relationship.

Along these lines, it has been reported that the closer the relationship, such as being single versus being married or in committed relationships, reduces the report of jealousy (Demirtaş and Dönmez, 2006). In the case of dependence, research found its association primarily with reactive jealousy (Rydell and Bringle, 2007). This may be because this type of jealousy depends on specific contextual factors (Buunk, 1997), rather than being measured solely by an individual’s perception of their subjective experience.

Our interpretation of love based mainly on interpersonal dependence can be viewed as romantic love, without triggering the feelings of real loss or a potential threat commonly experienced in jealousy. But, as research since Bowlby’s (1982) seminal work predicts and supports across time, jealousy is a response strongly related to attachment (Richter et al., 2022). And it is anxious attachment that appears to capture the affective traits that most likely mobilize jealousy.

Drawing on the conceptualization of romantic relationships as a collaborative effort, it has been proposed that members of a dyad face incentives, in evolutionary terms, where resources invested increase the individual fitness of both partners. Common resources can be viewed as benefits resulting from the cooperation with each other (Buss, 2003). The basic idea is that the resources of the couple together are greater than the resources of the individuals alone (Kaplan and Lancaster, 2003; Conroy-Beam et al., 2015).

In general, within this framework, jealousy could resolve discrepancies between actual and expected investment in a relationship, and love plays a role in motivating individuals to maintain commitment, invest time and psychological resources on the other, and allocating reproductive resources necessary for adaptive fitness in cooperative relationships (Conroy-Beam et al., 2015).

One important limitation of the current research is the evaluation of love and jealousy as trait measures, as well as the reliance on a single self-report question about how jealous an individual is. For future research it would be ideal to include an actual relationship jealousy and love scales.

Finally, it would be recommended that further research specifically focus on differentiating the potential protective function of jealousy in regard to a specific partner and the levels of love or interpersonal dependence between them. It would also be expected that manipulating or varying relationship satisfaction should have an effect on jealousy, and possibly on love as well.

## Data availability statement

The raw data supporting the conclusions of this article will be made available by the authors, without undue reservation.

## Ethics statement

The studies involving humans were approved by Comite de Etica, Universidad de Santiago de Chile. The studies were conducted in accordance with the local legislation and institutional requirements. The participants provided their written informed consent to participate in this study.

## Author contributions

AF and MB contributed to conception and design of the study. AF, MB, and BC organized the database and conducted the statistical analysis. BC and YA wrote sections of the manuscript. All authors contributed to manuscript revision, read, and approved the submitted version.
